# Case Report: A New Surgical Approach to Cervical Hyperlordosis

**DOI:** 10.5812/traumamon.16023

**Published:** 2014-08-01

**Authors:** Ebrahim Ameri, Hasan Ghandhari, Naveed Nabizadeh

**Affiliations:** 1 Department of Orthopedic Surgery, Shafa-Yahyaeian Hospital, Iran University of Medical Sciences, Tehran, IR Iran; 2 Shohada Hospital, Shahid Beheshti University of Medical Sciences, Tehran, IR Iran

**Keywords:** Pediatrics, Abnormalities, Posture

## Abstract

**Introduction:**

Cervical hyperlordosis is a rare pediatric deformity leading to gaze and postural disturbances. The cornerstone of treatment consists of spinal manipulative therapy (SMT) combined with positional traction.

**Case Presentation:**

We report a new surgical approach in a 7-year-old female patient suffering from stiff cervical hyperlordosis, desiring to correct forward head posture as well as gaze disturbance. The patient had a chief complaint of restricted range of motion of the neck for the past 4 years. Posture examination revealed several abnormalities, including apparent thoracic hump with shifting to right side, slight elevation of the right shoulder, with back pain. She also had difficulties performing her assignments. Radiological investigations revealed a 95˚ cervical lordosis and forward head posture (FHP) assessed by two separate measurements. There was no considerable response to conservative treatment, (which included 30 sessions of SMT combined with positional traction). Consequently, she underwent radical resection of cervical paraspinal muscles, followed by halo traction. She was discharged with a halo-vest. Specific instructions for home exercise were provided to the patient. Post-trial radiographs showed a reduction of cervical lordosis to 51˚ and a reduction in FHP of 73 mm. The symptoms were alleviated at the end of the treatment.

**Discussion:**

This new approach appeared to correct postural abnormalities, and had an obvious positive effect on the patient’s chief complaint.

## 1. Introduction

The adverse effects of cervical hyperlordosis are documented in only a few reports. Cervical hyperlordosis may put increased stress on the posterior joint system, potentially leading to neck pains and other posterior joint problems (1). One of important consequences of cervical hyperlordosis is gaze disturbance (2). Specific rehabilitation techniques have been described, showing a reasonable efficacy on restoring cervical lordosis (3, 4). However, their efficacy in treating hyperlordosis has not been confirmed.

Due to the reported failure of spinal manipulative therapy (SMT) alone, as a treatment for cervical hyperlordosis, we recommend a surgical technique to correct physical posture and improve gaze disturbance.

## 2. Case Presentation

A 7 year-old girl presented with a chief complaint of gradual restriction of cervical range of motion. Four years ago, her parents discovered the initial limited rotation of neck with subsequent limited flexion and eventual cervical hyperlordosis deformity. As she started school homework, the downward gaze difficulties became more prominent. The family asked for a major medical intervention when they found that she could not perform her school assignments. She had undergone 30 sessions of stretching exercise and positional traction, without satisfactory results.

The preliminary physical exams revealed thoracic hump, elevated right shoulder, extremely limited cervical flexion (active and passive); lateral cervical bending less than 10˚ and mild back pain due to awkward positions. The only finding in the physical exam of extremities was limited external bilateral hip rotation at less than 20˚. Absence of knee or hip flexion contracture, “W” sitting position, normal neurologic exam and lack of limping were other findings. A palpatory examination revealed a significant amount of bilateral muscle thickness and hardness in the paraspinal musculature about the C1 - C7 levels. Physical examination revealed no abnormal sensory, motor, or reflex findings. Bilaterally apparent lack of cervical rotation was recognized. The radiological examination revealed a 95˚ cervical hyperlordosis, significantly higher than the normal range of 34 - 42˚ (2, 5). The cervical vertebrae bony architecture was normal. We found thoracic scoliosis of about 20 degrees at T4-T10 level, which required no surgical interventions.

The forward head posture (FHP), when measured from the sella turcica down to the anterior portion of the C4 disc, as outlined by Kapandji (6), measured -60 mm. This value should be zero, according to Kapandji. Due to the cervical hyperlordosis, measurements of the FHP were also performed, using a vertical line from the posterior-superior corner of the C2 vertebral body down to the posterior inferior corner of the vertebral body of C7. This method is outlined by Harrison et al. (3, 5). The initial measurement, using this method, was 10 mm, which was in the normal range. In addition, we also measured the C7 sagittal tilt by drawing a line parallel to the inferior end plate of C7, and measuring that line against a line constructed horizontally. Initially, this angle measured 40˚ (2). The magnetic resonance imaging (MRI) study was not conclusive. Investigation by CT scan did not reveal any congenital vertebral abnormalities. Electromyography (EMG) and nerve conduction studies did not help to differentiate any spastic or neuromuscular underlying disease. There was no considerable response to conservative treatment including 30 sessions of SMT combined with positional traction. Botulinum injection was not recommended due to the lack of any spastic disorders. Therefore, she underwent surgical radical resection of cervical paraspinal muscles, followed by halo-traction. She was discharged with a halo-vest. The purpose of halo application was to prevent the recurrence of deformity. Specific instructions for home exercises were provided to the patient. Apparently, halo devices can be used safely in children as young as 1 year old. The advantages of halo-vest in comparison with other cervical orthosis are: 1. properly applied custom halo-vest avoids the skin breakdown complications; 2. the potential noncompliance of children with removable collars and orthosis favors the use of halo devices.

Post-trial radiographs showed a reduction of the cervical lordosis down to 51˚ and a reduction in FHP to 73 mm. Patient symptoms were significantly alleviated by the end of the aforementioned treatment.

### 2.1. Procedure

General anesthesia was administrated in supine position. After administration of muscle relaxants, we did not achieve any evidence of cervical flexion. Then, we positioned the patient prone on a three-point head rest and avoid any excessive pressure on the eyes. After routine skin preparation, we attached the drapes to the neck with stay sutures. By a midline, longitudinal skin incision from the occiput to C7, the skin and subcutaneous tissues were undermined and paraspinal muscles were exposed. The trapezius was separated from underlying muscles by blunt dissection. The fascial sheath on the origins of the trapezius and rhomboid muscles was freed from the spinous processes by a sharp dissection. Afterwards, the origins of the splenius and semispinalis muscles were stripped from the occiput. About 3 cm of the proximal parts of the last muscles were excised for pathologic study. By gentle flexion of the neck intraoperatively, we were able to evaluate the adequacy of the contracture release. The optimal release was revealed intraoperatively by chin-to-manubrium contact. Then, the midline fascia and nuchal raphe were repaired, while a drain was placed along the posterior aspect of the spine to prevent the formation of hematoma. The skin was closed in multiple layers. After dressing, the patient was placed in supine position and a halo ring was applied by introducing the pins in the appropriate locations. The initial traction was started by 2 lbs. and gradually increased to 6 lbs. over 2 weeks. The pathology report of the muscle specimen was normal. She was discharged from the hospital with a halo cast for 6 weeks. After halo ring removal, instructions for stretching exercises were provided.

**Figure 1.**
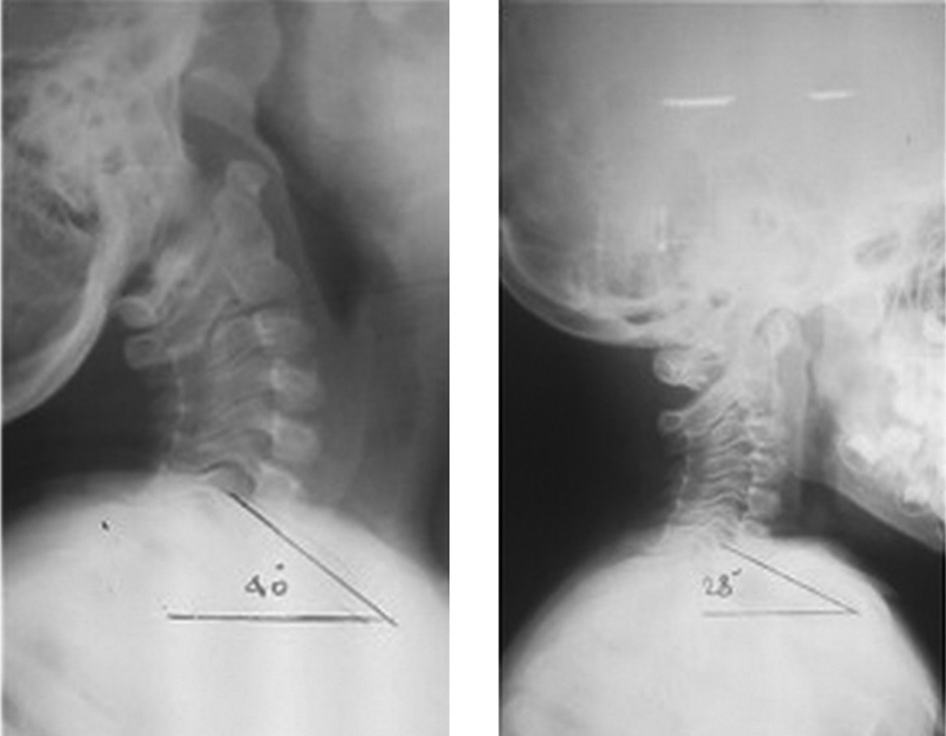
C7 Sagittal Tilt

**Figure 2.**
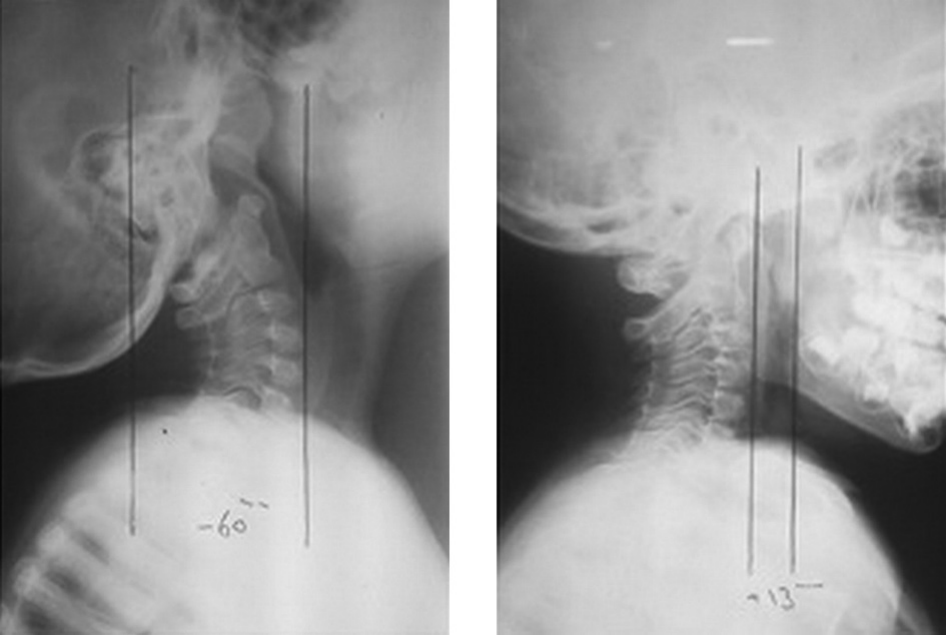
Forward Head Posture, When Measured From the Sella Turcica Down to the Anterior Portion of the C4 Disc. Preoperative and postoperative X-ray.

The postoperative X-ray study showed that cervical lordosis had been reduced from 95˚ to 51˚, which falls closer to the normal range. In addition, the C7 sagittal tilt was also reduced from 40˚ to 28˚. The FHP, when measured from the sella turcica, changed from -60 mm to 13 mm. However, when it was measured from the posterior superior C2 body corner, the FHP was reduced from 10 mm to 6 mm. All changes are illustrated in Figures 1 and 2.

## 3. Discussion

Apparently, regardless of its etiology, cervical hyperlordosis has an adverse effect on gaze and posture (2). When there is no significant response to conservative treatment, including SMT combined with positional traction, we suggest the radical resection of cervical paraspinal muscles followed by the application of halo-traction. The present article introduces a new procedure to essentially correct head balance on the trunk and partially the cervico-thoracic sagittal balance. This specific technique results in considerable recovery as well as symptomatic improvement. Postoperative downward gaze and neck flexion showed a dramatic improvement. Post-trial radiographs showed a reduced cervical lordosis down to 51˚. In postsurgical radiographs, the C7 vertebra sagittal tilt approximated the horizontal line. It was suggested by Takeshima et al. (7) that the position of the C7 vertebra is predictive of static alignment of the spine. Their opinion is that the static position of the C7 vertebra is associated with a more upright cervical spine (7). A consistent result was the postoperative correction of thoracic kyphosis from 54˚ to 40˚.

In the present case, FHP measurement based on sella turcica position revealed anterior displacement of about 73 mm postoperatively. However, when FHP was measured by C2 body corner, postoperative displacement was only 4 mm. Based on this issue, it may be proposed that following the technique of paraspinal muscle resection, most of the cervical hyperlordosis correction is secondary to the improvement of head balance on the trunk. Furthermore, the correction of the alignment of C2 compared to C7 is not significant by this procedure. In other words, the major part of the sagittal realignment is achieved by remarkable correction of C7 sagittal tilt (about 12˚) which improves the overall alignment of the spine. However, in comparison with C7, the relocation of C2 represents only a minor part of the realignment.

The last follow up 2 years postoperatively and did not show evidence of recurrence or visual gaze problems. This approach appeared to correct postural abnormalities, and had a remarkable positive effect on the patient’s chief complaint. Paraspinal muscle resection technique for treating cervical hyperlordosis needs to be applied in a greater number of similar cases to determine its true indications and effectiveness.
